# Carotid body tumor: retrospective analysis on 22 patients

**DOI:** 10.1590/1516-3180.2014.1323452

**Published:** 2014-04-14

**Authors:** André Luís Maion Casarim, Alfio José Tincani, André Del Negro, Camila Guimarães Aguiar, Renato Ventura Fanni, Antonio Santos Martins

**Affiliations:** I MD. Attending Physician, Head and Neck Surgery Service, Department of Surgery, School of Medical Sciences, Universidade Estadual de Campinas (Unicamp), Campinas, São Paulo, Brazil; II MD, PhD. Associate Professor, Head and Neck Surgery Service, Department of Surgery, School of Medical Sciences, Universidade Estadual de Campinas (Unicamp), Campinas, São Paulo, Brazil

**Keywords:** Paraganglioma, Neoplasms, Carotid body tumor, Head and neck neoplasms, Carotid artery diseases, Paraganglioma, Neoplasias, Tumor do corpo carotídeo, Neoplasias de cabeça e pescoço, Doenças das artérias carótidas

## Abstract

**CONTEXT AND OBJECTIVE:**

Carotid body tumors, or chemodectomas, are the most common head and neck paragangliomas, accounting for 80% of the cases. They may present minor symptoms; however, they deserve special attention in order to achieve accurate diagnosis and adequate treatment. The objectives of this study were to show the approach towards chemodectomas and evaluate the complications of the patients treated surgically without previous embolization.

**DESIGN AND SETTING:**

Retrospective study on chemodectomas followed up at the Head and Neck Surgery Service, Department of Surgery, Unicamp.

**METHODS:**

Twenty-two patients were evaluated between 1983 and 2009. The diagnosis was based on clinical findings and imaging methods. The epidemiological characteristics, lesion characteristics, diagnostic methods, treatment and complications were analyzed.

**RESULTS:**

The paragangliomas were classified as Shamblin I (9%), II (68.1%) and III (22.7%). Angiography, magnetic resonance imaging and computed tomography confirmed the diagnosis in 20 patients (90.9%). Five (22.7%) had significant bleeding during the surgery, while four (18.1%) had minor bleeding. Four patients (18.1%) developed neurological sequelae. Seven (31.8%) needed ligatures of the external carotid artery. Three patients (13.6%) underwent carotid bulb resection. The postoperative follow-up ranged from 3 months to 14 years without recurrences or mortality.

**CONCLUSIONS:**

In our experience and in accordance with the literature, significant bleeding and neurological sequelae may occur in chemodectoma cases, particularly in Shamblin III patients. The complications from treatment without previous embolization were similar to data in the literature data, from cases in which this procedure was applied prior to surgery.

## Abstract (pt)

**CONTEXTO E OBJETIVO:**

O tumor de corpo carotídeo, ou quimiodectoma, é o paraganglioma mais comum em cabeça e pescoço, com aproximadamente 80% dos casos. Pode apresentar poucos sintomas; no entanto, necessita atenção especial para o diagnóstico e tratamento adequado. Os objetivos deste estudo são mostrar a abordagem do quimiodectoma e avaliar as complicações nos pacientes tratados cirurgicamente sem embolização prévia.

**TIPO DE ESTUDO E LOCAL:**

Estudo retrospectivo de quimiodectomas acompanhados pelo Serviço de Cirurgia de Cabeça e Pescoço, Departamento de Cirurgia, Unicamp.

**MÉTODOS:**

Vinte e dois pacientes foram avaliados entre 1983 e 2009. O diagnóstico foi baseado em achados clínicos e métodos de imagens. Foram analisados aspectos epidemiológicos, características das lesões, métodos diagnósticos, tratamento e complicações.

**RESULTADOS:**

Os paragangliomas foram classificados em Shamblin I (9%), II (68,1%) e III (22,7%). Angiografia, ressonância nuclear magnética e tomografia computadorizada confirmaram o diagnóstico em 20 pacientes (90,9%). Cinco (22,7%) tiveram sangramento significativo durante a cirurgia, enquanto quatro (18,1%) tiveram sangramento mínimo. Quatro pacientes (18,1%) tiveram sequelas neurológicas. Sete (31,8%) necessitaram de ligadura da artéria carótida externa. Três (13,6%) foram submetidos a ressecção do bulbo carotídeo. O acompanhamento variou de 3 meses a 14 anos, sem recorrências ou óbitos.

**CONCLUSÕES:**

Em nossa experiência e de acordo com a literatura, sangramentos significativos e sequelas neurológicas podem ocorrer nos quimiodectomas principalmente em pacientes Shamblin III. As complicações do tratamento sem embolização prévia foram similares aos relatos observados na literatura nos quais se aplicou tal procedimento antes da cirurgia.

## INTRODUCTION

These tumors are often benign, presenting slow growth,^2^ and are located in the upper cervical region. There is no evidence of gender predominance. They may only present minor symptoms, commonly only a pulsatile mass in the neck. However, they can cause local discomfort, dysphagia, hoarseness, stridor, vertigo and paralysis of the cranial nerves, and sometimes attain large volumes (some authors have described tumors as large as 10 cm).^1^

They can be divided into tumors with either sporadic or familial traits. In the sporadic type, bilaterality has been reported in 5 to 10% of the cases.^3^ The familial form is manifested through a dominant autosomal gene, and in these cases, bilaterality may reach up to 30%.^4^ Recent studies have described a mutation along germinative lines, which may explain the etiology, through identification of six specific genes (RET, VHL, NF1 and subunits of SDH).^4^ The hereditary form is mostly correlated with mutations in the SDHD gene.^5^ ^,^ ^6^

The incidence in the general population is difficult to estimate. However, according to Rodriguez-Cuevas,^7^ the incidence in the general population is about 0.01%. Since 1969, when Árias-Stella demonstrated carotid body enlargement in high-altitude Peruvian populations (due to the low oxygen pressure), it has been known that chronic hypoxia is an important factor in the etiology of this neoplasm.^8^ The malignant potential is about 6% and the criteria are based on cell atypia, mitosis, local invasions and, especially, by the presence of metastasis.^9^ ^-^ ^11^

The diagnosis is based on clinical history, physical examination, imaging methods (such as ultrasound, computed tomography and magnetic resonance) and vascular evaluations (including angiography, tomographic angiography and magnetic resonance angiography).^12^ ^,^ ^13^ In this context, based on the Shamblin classification, Arya et al. used magnetic resonance imaging to pre-classify carotid body tumors before surgical treatment.^14^ This could improve preoperative management and reduce the complication rate.^9^

The best treatment for carotid body paragangliomas is a surgical approach. The literature shows that patients may or may not undergo prior embolization. Some authors have justified using embolization because it reduces complications such as bleeding or neurological sequelae,^15^ although this procedure is not without its own inherent complications.^16^

## OBJECTIVES

The objectives of this study were to present an academic institution's experience of dealing with carotid body paragangliomas, such as the diagnostic methods, complementary examinations and treatment, and to show the possible complications that may occur over the natural evolution of the disease or in relation to surgery without previous embolization.

## METHODS

Bleeding was measured in our study and was classified as minimal (less than 200 ml), moderate (ranging from 200 to less than 1000 ml) and significant (over 1000 ml), according to the need for blood transfusion. Thus, when the bleeding was minimal, blood transfusion was not needed. In cases of moderate bleeding, one or two red blood cell units were infused. Finally, when the bleeding was significant, more than two red blood cell units were infused.

The tumors were divided by means of the Shamblin classification,^8^ according to the involvement of the carotid wall and difficulty of surgical resection. Shamblin I was reserved for tumors that were relatively small and easy to resect, with minimal or no adherence to the carotid wall. Shamblin II consisted of tumors with partial involvement of the carotid wall, and Shamblin III completely involved the carotid bifurcation. 

**Figure 1:**
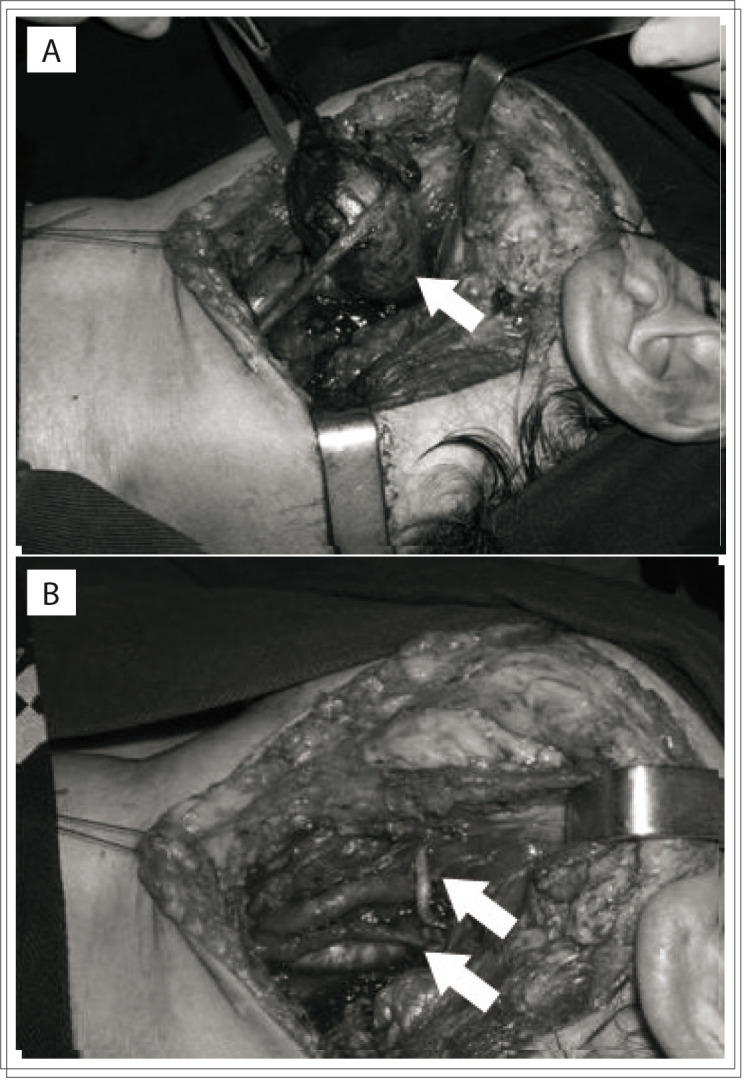
Tumor mass dissected at the carotid bifurcation. (A) Arrow shows carotid body tumor. (B) Preserved carotid arteries (arrows) after resection of carotid body tumor

All the patients underwent surgical treatment, without embolization or prior radiotherapy. The resection was complete in all patients, with a sub-adventitial avascular approach (Figure 1).

## RESULTS

**Figure 2:**
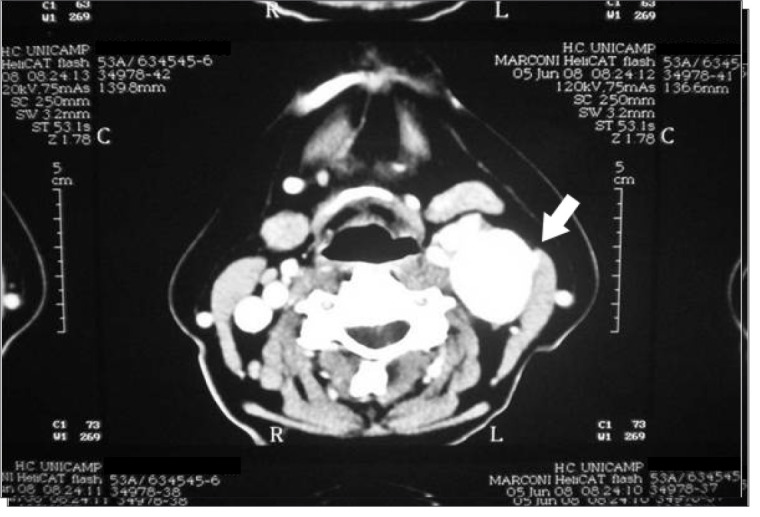
Computed tomography showing left parapharyngeal tumor (arrow)

Our study included two patients with Shamblin I (9%), 15 with Shamblin II (68.1%) and five with Shamblin III (22.7%). Fourteen patients (63.6%) were female. The patients' ages ranged from 23 to 77 years (average of 43 years) and all of them had an asymptomatic cervical mass at the initial presentation. The mass diameter ranged from 3 to 8 centimeters (mean of 4.3 cm). Two cases (9%) were bilateral, without any other signs or symptoms. The length of history ranged from 8 months to 13 years (average of 5 years), with the largest tumor occurring in the patient with the longest history. Six patients (27.2%) had undergone surgery for a biopsy or resection in other services, and all procedures were incomplete and interrupted because of excessive bleeding. In our service, the diagnosis was suspected preoperatively in all patients, with two exceptions (9%). Consequently, 91% were managed with predefined surgical planning. Angiography was performed on 15 patients (68.1%), cervical CT scan on 11 (50%), cervical ultrasonography on nine (40.9%), and MRI on five patients (22.7%) (Figures 2 **to 4**). The two unsuspected cases, which underwent ultrasonography, had a preoperative diagnosis of branchial cleft cyst and undefined adenopathy. These cases were classified subsequently as Shamblin II and did not have any postoperative sequelae or complications.

**Figure 3:**
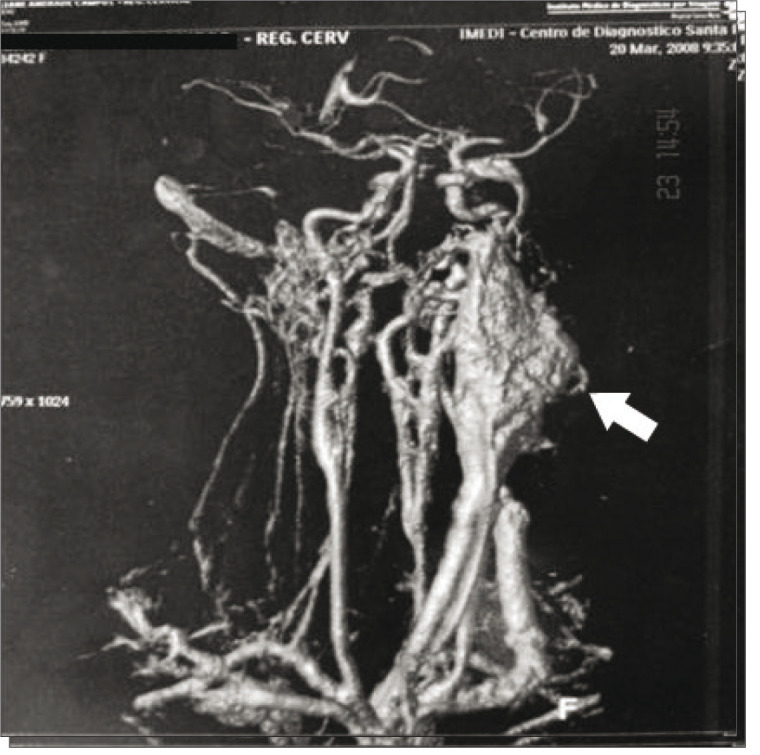
Magnetic resonance angiography with three-dimensional reconstruction, showing a vascular mass at the carotid bifurcation (arrow)

**Figure 4:**
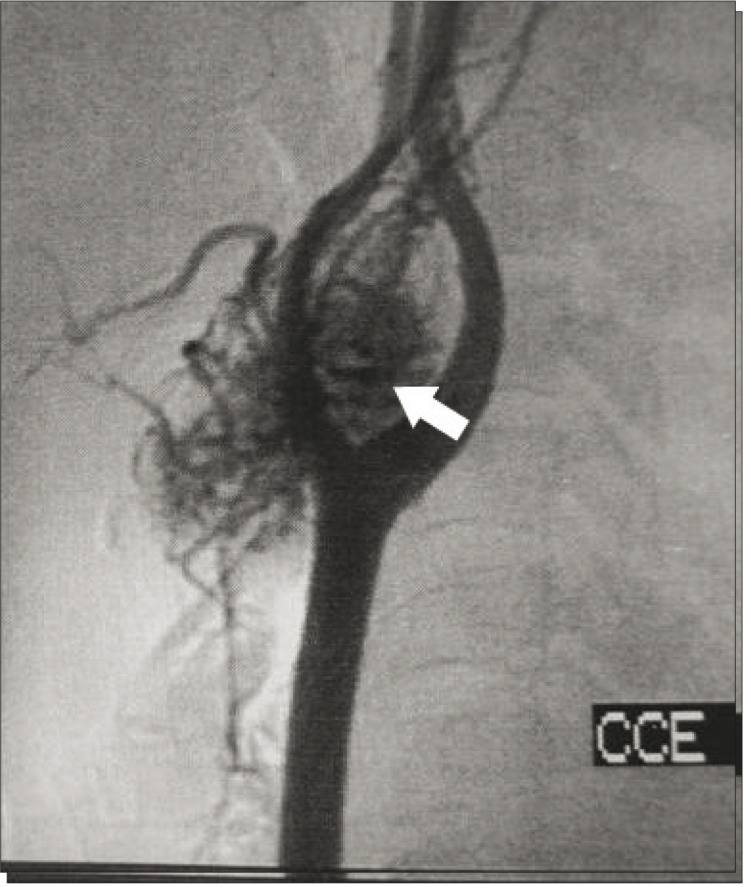
Angiography showing vascular mass at the carotid bifurcation (arrow)

All the imaging methods with arterial evaluations (angiography, CT and MRI) were diagnostic for the patients who underwent these examinations, with no wrong preoperative diagnoses. 

Ultrasonography was performed on nine patients (40.9%), and was helpful in diagnosing seven cases (77.7%). However, this examination (particularly when there was no Doppler study) suggested the possibilities of branchial cyst and adenomegaly in two cases that had wrong preoperative diagnoses.

Fine needle aspiration (FNA) was performed on five patients (35.7%), and was suggestive of the diagnosis in four cases (80%). In one of the patients, who also underwent ultrasonography without a Doppler study, FNA erroneously diagnosed a branchial cyst. 

During the surgical procedure, five patients (22.7%) presented significant bleeding (over 1000 ml). Three of these patients were Shamblin III and two were Shamblin II, and the former had incorrect preoperative diagnoses. 

Among the five Shamblin III patients, three (60%) had postoperative neurological sequelae. One patient had a sympathetic chain deficit that evolved to Horner's syndrome. The second had a vagus nerve deficit and evolved with hoarseness and the third patient had a transient ischemic attack that resolved completely one day after the symptoms, without late neurological sequelae.

Twelve patients (54.5 %) had moderate bleeding (ranging from 200 to less than 1000 ml). Of these, nine patients (75%) were Shamblin II and three (25%) were Shamblin III. In this group, one patient (8.3%) who was classified as Shamblin II evolved with unilateral hypoglossal nerve deficit (close contact with the tumor). 

In the five remaining patients (22.7%), the intraoperative bleeding was considered to be minimal (up to 200 ml). These patients were all Shamblin I or II and none had neurological sequelae.

In nine of our patients (40.9%), some form of vascular procedure was needed. Seven patients (31.8%) needed external carotid ligation and three of these needed en-block resection of the carotid bulb. In two cases, there was a need for intraluminal vascular shunts for arterial reconstruction. In such cases, the possibilities of distal clot formation and stroke need to be borne in mind, and in one of our patients, this was managed by using distal artery aspiration, with a flexible arterial catheter and a 10 ml syringe. Through this, the clot was removed with immediate reflux of the distal internal carotid artery, without sequelae. The vascular reconstruction was done using a saphenous vein graft in one patient, and end-to-end anastomosis between the common and the internal carotid artery in the other two patients. Intraluminal shunt was used in these cases to prevent stroke. 

**Graph 1:**
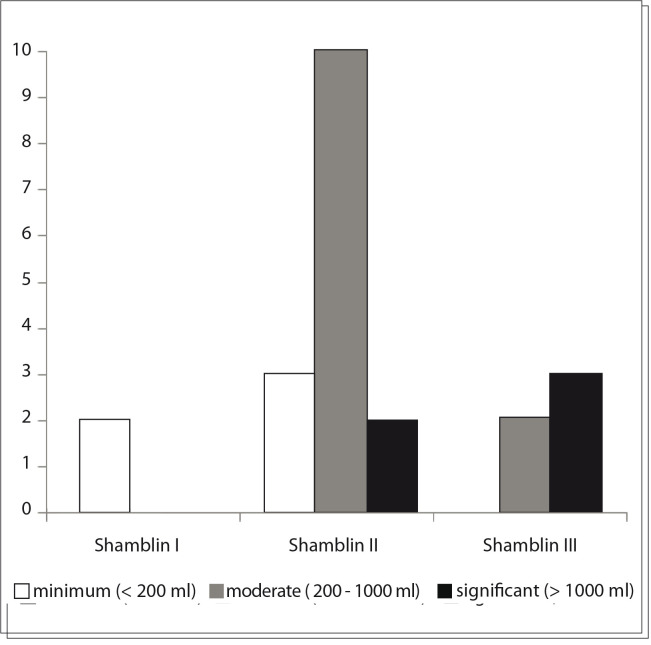
Intraoperative bleeding according to Shamblin classification

Graph 1 presents the intraoperative bleeding according to the Shamblin classification, and shows the relationship between the bleeding and higher Shamblin classification. Considering all the patients, the bleeding ranged from 50 ml to 2,000 ml, with a mean of 667 milliliters.

**Graph 2:**
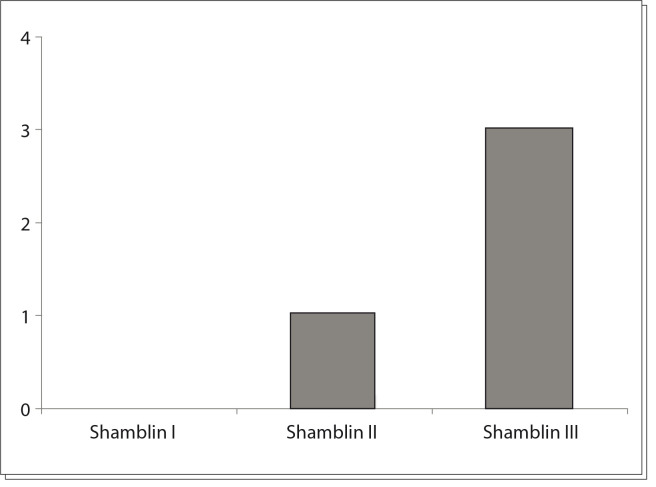
Neurological sequelae according to Shamblin classification

In total, four patients (18.1%) had neurological sequelae (Graph 2). One developed Horner's syndrome; one presented permanent deficits of the IX, X and XII cranial nerves; another had an isolated permanent XII nerve deficit; and the last had a transient ischemic attack.

Another important complication was intraoperative cardiac arrest due to the carotid bulb manipulation. This affected one patient and was managed immediately with external cardiac massage and infusion of adrenaline and atropine. The cardiac activity returned successfully less than one minute after the intercurrence, and the patient evolved without sequelae. 

Bilaterality was found in two patients (9%), which was managed surgically in two different settings. One of these patients had an association with chronic hypoxia (severe cardiac valve disease, interventricular communication and a persistent arterial channel). He exhibited frequent hypotension during the postoperative period, possibly secondary to slow adaptation to hypoxia, after resection of the carotid chemoreceptors. The other patient presented cardiac valve disease from rheumatic fever.

The postoperative follow-up ranged from 3 months to 14 years, averaging 5.1 years, with no recurrences and no mortality, but with the abovementioned morbidity.

## DISCUSSION

The incidence of this tumor is greater at higher altitudes, due to the chronic hypoxia.^7^ There have been several reports in the literature mentioning absence of significant predominance between the genders, although our series showed predominance of females (63.6%). These tumors are difficult to diagnose because of their rarity and few manifested symptoms. The paramount clinical characteristic is a pulsatile cervical mass. As reported in the literature, the diagnosis can be confused even with benign conditions, including congenital branchial cysts^13^ or other benign conditions. Thus, as pointed out in the literature, a pulsatile mass near the carotid bulb, in the absence of any suggestion of metastatic disease, should raise the possible diagnosis of chemodectoma and should be followed by specific arterial imaging studies.

FNA was helpful for the diagnosis in the cases of four patients in our study. However, this procedure may be dangerous and performing it is controversial because of the risk of bleeding.^2^

Ultrasonography and CT scans can be very helpful for the preoperative diagnosis and may show enlargement of the bifurcation and hypervascularized tumors. Some authors have reported that ultrasonography with Doppler flowmetry can help surgeons in the initial approach.^17^ However, CT scans or magnetic resonance imaging are better for identifying the dimensions and anatomical correlations of the tumor.^18^ The main diagnostic tool is arterial studies such as contrast angiography, computed angiotomography or magnetic resonance angiography (MRA), thereby confirming or not confirming the suspected diagnosis. The MRA seems to be preferable nowadays.^14^

In the literature, the best treatment is considered to be surgical, with dissection of the tumor in the sub-adventitial avascular plane of the artery.^12^ Large tumors (Shamblin II and III) may require vascular procedures, including repairs, sutures and resections of the arterial segments.^19^ At times, it may be necessary to sacrifice the external carotid artery, perform anastomosis between the internal and common carotid arteries or undertake vascular reconstruction with grafts.^11^ ^-^ ^13^ ^,^ ^20^ ^,^ ^21^ Some authors have described use of a stent inserted into the internal carotid artery before the surgery, in cases presenting as Shamblin III with complete involvement of this artery.^22^ Occurrences of technical complications in anastomoses between the common and internal carotid arteries after resection of the carotid body have also been reported in the literature. These cases evolved with several hematomas and, in some cases, with stroke caused by deficits of cerebral irrigation.^8^ ^,^ ^13^ ^,^ ^23^ This complication did not occur in our series.

In patients with bilateral tumors, obviously, the procedures should not be done at the same time, but spaced out one from another, especially because of possible vascular or cranial nerve lesions.^8^ Conservative treatment should be reserved for patients who are not suitable for surgery, such as clinically unstable patients, extremely old patients or those with the certainty of stroke. Some authors have suggested that radiotherapy could be used, but this is not recommended by most institutions. Furthermore, studies have shown that this therapeutic method is ineffective in these tumors.^3^ ^,^ ^24^ ^-^ ^28^

Embolization is another therapeutic method, usually done prior to the surgical procedure, as an attempt to decrease intraoperative bleeding.^21^ ^,^ ^29^ However, this demands specialized skill and also has the risk of possible significant vascular complications and cranial nerve deficits. In our service, the patients were treated without preoperative embolization. In the literature, it is mentioned that vascular complications and cranial nerve deficits can occur in about 33% of the patients.^1^ ^,^ ^11^ A famous study at the Mayo Clinic evaluated patients' complications compared between three periods of that institution's surgical history. It was concluded that, despite the decreases in vascular complications and mortality over the course of time, through better diagnostic methods and surgical technologies or skills, the rate of neurological injuries was not statistically different between the three periods studied, spanning a total of fifty years.^30^ In our series, cranial nerve deficits occurred in two patients, involving the IX, X and XII pairs. We had another patient who evolved with sympathetic chain palsy. Power et al. demonstrated that preoperative embolization may have some benefits for the surgical approach, especially relating to surgical bleeding, but the patients that they followed up did not present any differences in definitive neurological sequelae.^16^

Although our series did not have any cases of malignant chemodectoma, it is important to mention that the malignant potential has been reported to be around 2-6 % in the literature, most frequently with metastasis to regional lymph nodes.^8^ ^,^ ^23^ Some authors have prescribed chemotherapy for metastasized cases.^31^

Finally, although we did not have any cases of mortality, the literature mentions a mortality rate of less than 2%.^1^

## CONCLUSIONS

Complementary imaging study methods such as ultrasonography, computed tomography, magnetic resonance or, especially, arteriography need to be included in evaluations today, in order to analyze the involvement of the carotid artery and to program the surgical treatment for carotid body tumors.

This study, in agreement with the literature, demonstrated that significant bleeding and neurological sequelae may occur, and that this risk increases according to the Shamblin classification, particularly in Shamblin III patients. Even though preoperative embolization may be an important alternative, the complications observed in treatment without previous embolization were similar.
